# The Proteomic Landscape of the Suprachiasmatic Nucleus Clock Reveals Large-Scale Coordination of Key Biological Processes

**DOI:** 10.1371/journal.pgen.1004695

**Published:** 2014-10-16

**Authors:** Cheng-Kang Chiang, Neel Mehta, Abhilasha Patel, Peng Zhang, Zhibin Ning, Janice Mayne, Warren Y. L. Sun, Hai-Ying M. Cheng, Daniel Figeys

**Affiliations:** 1 Ottawa Institute of Systems Biology and Department of Biochemistry, Microbiology and Immunology, Faculty of Medicine, University of Ottawa, Ottawa, Ontario, Canada; 2 Department of Biology, University of Toronto Mississauga, Mississauga, Ontario, Canada; Charité - Universitätsmedizin Berlin, Germany

## Abstract

The suprachiasmatic nucleus (SCN) acts as the central clock to coordinate circadian oscillations in mammalian behavior, physiology and gene expression. Despite our knowledge of the circadian transcriptome of the SCN, how it impacts genome-wide protein expression is not well understood. Here, we interrogated the murine SCN proteome across the circadian cycle using SILAC-based quantitative mass spectrometry. Of the 2112 proteins that were accurately quantified, 20% (421 proteins) displayed a time-of-day-dependent expression profile. Within this time-of-day proteome, 11% (48 proteins) were further defined as circadian based on a sinusoidal expression pattern with a ∼24 h period. Nine circadianly expressed proteins exhibited 24 h rhythms at the transcript level, with an average time lag that exceeded 8 h. A substantial proportion of the time-of-day proteome exhibited abrupt fluctuations at the anticipated light-to-dark and dark-to-light transitions, and was enriched for proteins involved in several key biological pathways, most notably, mitochondrial oxidative phosphorylation. Additionally, predicted targets of miR-133ab were enriched in specific hierarchical clusters and were inversely correlated with miR133ab expression in the SCN. These insights into the proteomic landscape of the SCN will facilitate a more integrative understanding of cellular control within the SCN clock.

## Author Summary

The suprachiasmatic nucleus (SCN) serves as the master circadian pacemaker in mammals, coordinating the physiological responses of a myriad of peripheral clocks throughout the body and linking their rhythms to the environmental light-dark cycle. In this study, we interrogated the murine SCN proteome across the circadian cycle using stable isotope labeling by amino acids in cell culture (SILAC)-based quantitative mass spectrometry. Among 3275 identified proteins in the SCN, 421 displayed a time-of-day-dependent expression profile, 48 fit a circadian expression profile with a ∼24 h period, and a surprising number of proteins were ultradianly expressed. Nine circadianly expressed proteins were accompanied by transcripts that were also 24 h rhythmic, but with a significant time lag (>8 h) between the phases of peak mRNA vs. protein expression. A substantial proportion of the time-of-day proteome exhibited abrupt fluctuations at the anticipated dawn and dusk, and was involved in mitochondrial oxidative phosphorylation. Additionally, predicted targets of miR-133ab were enriched in specific hierarchical clusters and were inversely correlated with miR133ab expression in the SCN. Our study underscores the significance of post-transcriptional regulation, the surprising prevalence of ultradian protein expression, and the functional implications on mitochondrial energy metabolism.

## Introduction

Proper temporal organization of behavioral, physiological and biochemical processes and their synchronization with the environmental light/dark cycle are fundamental features of most organisms [1]. In mammals, the central pacemaker that coordinates this adaptive response to temporal cues resides in the suprachiasmatic nucleus (SCN) of the anterior hypothalamus [2], [3]. The SCN is uniquely positioned to receive light signals from the retina and to relay the time information to peripheral clocks via synaptic and humoral mechanisms. Both central and peripheral clocks use a series of autoregulatory transcription-translation feedback loops to drive cell-autonomous, circadian (∼24 h) rhythms of gene expression of core clock components as well as tissue-specific, clock-controlled outputs [1].

In order to gain a bird's eye view of circadian regulation, a number of gene expression profiling studies using microarrays have been done to examine the circadian transcriptome of the murine SCN and liver [4], [5]. However, numerous studies have shown that transcript levels are not necessarily reliable predictors of protein abundance, and thus of functional outcome [6]. A previous attempt at elucidating the circadian proteome, using two-dimensional differential gel electrophoresis (2D-DIGE) combined with mass spectrometry (MS), identified 34 rhythmically expressed proteins within the SCN [7]. Until now, the small size of the SCN, and the limited amounts of protein that can be extracted from it, has posed a significant challenge to acquiring accurate and comprehensive quantitative proteomics data. However, recent technological advances in MS-based quantitation, and our growing awareness of the importance of post-transcriptional regulation of circadian rhythms [8], encouraged us to re-evaluate SCN functions from a proteomic perspective. In our previous study, we employed the AutoProteome system in conjunction with spectral counting to identify 2131 unique proteins in the SCN, of which 387 were acutely up- or down-regulated following nocturnal light exposure [9].

In this report, we performed an unbiased interrogation of the SCN proteome over a 24 h cycle using an alternative MS approach: a centrifugal proteomic reactor (CPR) coupled with stable isotope labeling by amino acids in cell culture (SILAC)-based quantitation. Neuro2A murine neuroblastoma cells were used as the SILAC-labeled internal reference standard for murine SCN tissues, based on reports that numerous core clock genes are expressed in this cell line, and that serum shock can induce robust circadian oscillations of their transcripts [10], [11]. Furthermore, a previous study indicated >97% overlap between the proteomes of mouse whole brain and Neuro2A cells [12]. Our proteomics screen identified a total of 3275 unique proteins in the murine SCN, 421 of which displayed time-of-day-dependent expression profiles. Within this smaller subset, 48 proteins fluctuated in a circadian manner. Bioinformatics analyses of these 421 proteins highlight the potentially important role that post-transcriptional mechanisms such as miRNAs may play in shaping the final profile of the time-of-day proteome, and the orchestrated expression of multiple proteins involved in neurosecretory processes and mitochondrial oxidative phosphorylation within the SCN.

## Results

### Interrogation of the SCN Proteome

**Figure 1:**
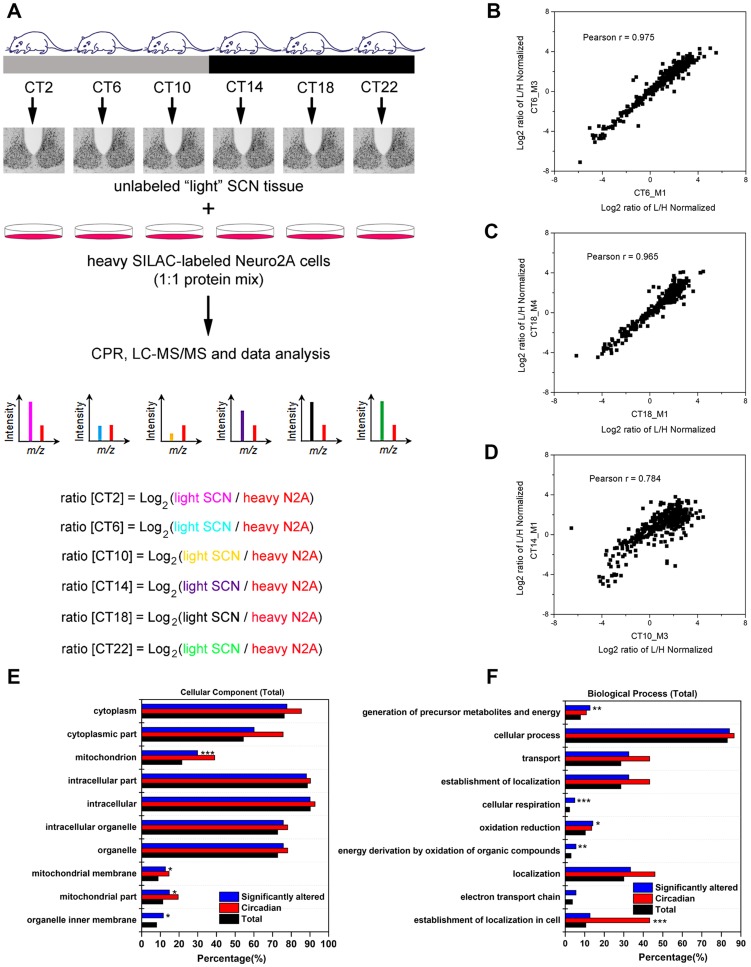
Global proteomic analysis of the murine SCN. (**A**) Schematic overview of the centrifugal proteomic reactor (CPR) coupled with SILAC-based quantification of the murine SCN proteome. Protein lysates (30 µg) extracted from the SCN of individual mice (n = 4 per CT; 6 CT in total) were mixed with equal quantities of protein lysates from heavy SILAC-labeled Neuro2A cells. The mixtures were processed by the CPR coupled with HPLC-ESI-MS/MS. A total of 3275 unique proteins were identified, of which 421 were significantly altered (time-of-day-dependent) in terms of protein expression levels during a 24-h cycle. (**B–D**) Biological replicates within a CT (B,C) showed a higher degree of correlation than samples harvested at different CTs (D). Scatter plots were plotted by logarithmized (Log_2_) normalized protein ratios (L/H) and the correlation coefficient (Pearson r) was calculated. (**E,F**) GO enrichment analysis by DAVID based on (E) total cellular component and (F) total biological process. The significantly altered (blue), circadian (red), and total SCN (black) proteomes were subject to enrichment analysis. All listed classifications were significant compared to the whole genome. Asterisks denote classifications that were significantly enriched compared to the SCN proteome (total). *p<0.05, **p<0.01, ***p<0.001 (Fisher's exact test).

To examine the murine SCN proteome, we stably entrained male C57Bl/6J mice to a 12 h light∶12 h dark (LD) cycle, and transferred them to constant darkness (DD) for two days. Starting at circadian time (CT) 2 on the third day of DD, we collected SCN tissues from four mice at 4 h intervals for a full circadian cycle (Figure 1A). SCN samples were processed individually to yield four independent biological replicates for each time point (4 mice per CT, n = 24 total mice). SCN protein lysates (30 µg) were mixed with equal quantities of lysates prepared from Neuro2A cells that had previously been cultured for >10 passages in heavy SILAC medium. Under these conditions, the Neuro2A proteome is estimated to be >98% heavy SILAC-labeled and thus useful as a spike-in reference standard. The CPR, with its superior recovery of hydrophobic membrane proteins compared with other approaches [13], was used for rapid protein preconcentration, derivatization, enzymatic digestion, and fractionation of the samples. Peptides were eluted from the CPR in ten fractions and analyzed by high performance liquid chromatography-electrospray tandem mass spectrometry (HPLC-ESI-MS/MS) in a total of 240 runs. From the raw mass spectrometric data, Maxquant and Andromeda identified 3275 protein groups with a false discovery rate (FDR) of 1% (Table S1). Out of these 3275 proteins, only 7 lacked a corresponding SILAC-labeled peak, indicating that these proteins are expressed in the SCN but not in Neuro2A cells (Table S1). As expected from recent proteomic studies [14], [15], our MS screen failed to detect any core clock proteins, likely due to their low abundance relative to the many cytoplasmic proteins which were detected. The raw dataset was further filtered for proteins that were identified by a minimum of two peptide ratio counts and where accurate quantification values were obtained in a minimum of 12 out of 24 independent samples. Downstream bioinformatics and statistical analyses were performed on this stringently filtered dataset of 2112 proteins (64.5% of total identified proteins), hereafter referred to as the SCN proteome (Table S2). High r values (between 0.83 and 0.97, Table S3) were obtained for the pairwise Pearson's correlation analysis of 24 measurements, indicating excellent reproducibility within our SCN proteome data.

**Figure 2:**
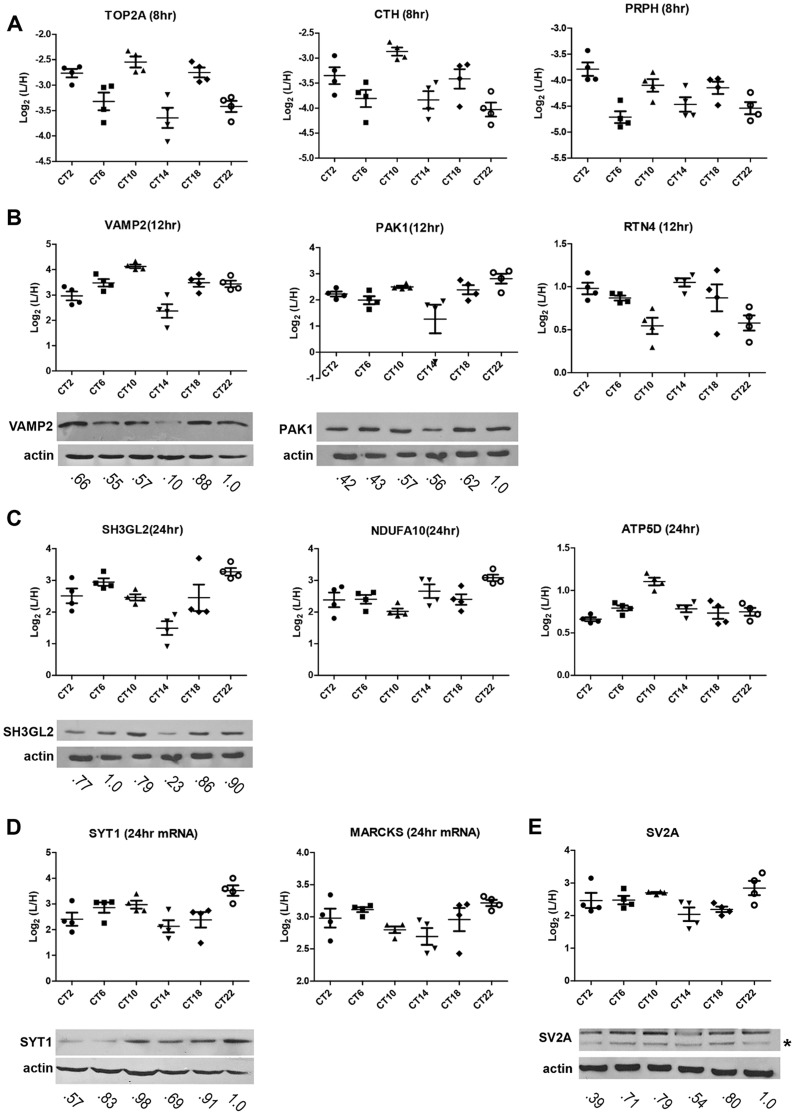
Robustness and validation of our SILAC-based SCN proteome. (**A–E**) Raw MS results of the temporal profiles of 12 time-of-day-dependent proteins including proteins that exhibit (A) 8 h, (B) 12 h, and (C) 24 h rhythms. (D,E) Time-of-day-dependent proteins, including those that are encoded by 24 h rhythmic transcripts (D), are also shown. Each graph was plotted with quantification values (Log_2_ (L/H)) in all 24 samples. The median value ± SEM of 4 biological replicate measurements for each CT (n = 4 per CT; 6 CT in total) is also shown. Time-of-day-dependent expression of SH3GL2, VAMP2, PAK1, SYT1 and SV2A were validated by Western blot (WB) analyses and presented below each MS plot. WB expression was analyzed at 6 CTs (2, 6, 10, 14, 18, 22). Actin was used as the loading control. Values below each blot represent the median relative abundance of the protein of interest, normalized to actin expression (n = 3 per CT). The asterisk (*) in panel (E) denotes the presence of a faster-migrating, non-specific band.

To identify proteins whose expression significantly fluctuated as a function of time-of-day, we subjected the SCN proteome to an analysis of variance. ANOVA revealed that 421 proteins (i.e., 20% of the SCN proteome) exhibited statistically significant (p<0.05) alterations in abundance across the 24 h cycle. This significantly altered proteome, hereafter referred to as the time-of-day proteome, was evaluated for reproducibility using pairwise Pearson's correlation analysis of the 24 samples (Table S4). The r values between biological replicates at a specific CT were extremely high (Figures 1B and 1C) compared with the lower r values observed between samples of different CTs (Figure 1D), indicating a high degree of reproducibility within biological replicates. As a second independent measure of variability, we calculated the relative standard deviation (RSD) for all proteins at every CT. The median RSD for the time-of-day proteome was 16%, compared with 11% from a previous study of the circadian hepatic proteome [14]. Considering that their study [14] utilized single pooled tissue samples per CT per 24 h cycle (2 cycles were analyzed), while ours uses unpooled samples from 4 animals per CT, an RSD of 16% reflects an acceptable level of variability within our dataset. Lastly, the temporal profiles of 12 proteins (Figure 2) with quantification values in all 24 samples also showed a higher degree of correlation within a CT than between CTs. The expression of five of these proteins—endophilin A1 (SH3GL2), synaptobrevin 2 (VAMP2), serine/threonine-protein kinase PAK 1 (PAK1), synaptotagmin 1 (SYT1), and synaptic vesicle glycoprotein 2A (SV2A)—was evaluated by Western blot (WB) analysis using independent batches of SCN tissues (Figure 2). In all cases, the WB results correlated well with our MS-based quantification (Pearson's coefficients = 0.78 [SH3GL2], 0.69 [VAMP2], 0.75 [PAK1], 0.77 [SYT1], 0.66 [SV2A]).

Next, the time-of-day proteome was subjected to Gene Ontology (GO) analysis by DAVID [16] in order to investigate its biological relevance. Relative to both the SCN proteome and the entire mouse genome (by DAVID), the time-of-day proteome was significantly enriched for GO total cellular components that were classified as mitochondrion or mitochondrial membrane (Fisher's exact test, p<0.05) (Figure 1E). Additionally, several metabolic pathways including generation of precursor metabolites and energy, oxidation reduction, energy derivation by oxidation of organic compounds, and cellular respiration were significantly enriched in this dataset based on GO total biological process analysis (Figure 1F). A more in-depth examination using GO FAT analysis, which filters out very broad GO terms based on a measured specificity of each term, confirmed that a significant portion of the time-of-day proteome was associated with the mitochondrion, energy generation and consumption, and hydrogen ion transmembrane transporter activity (Figure S1).

**Figure 3:**
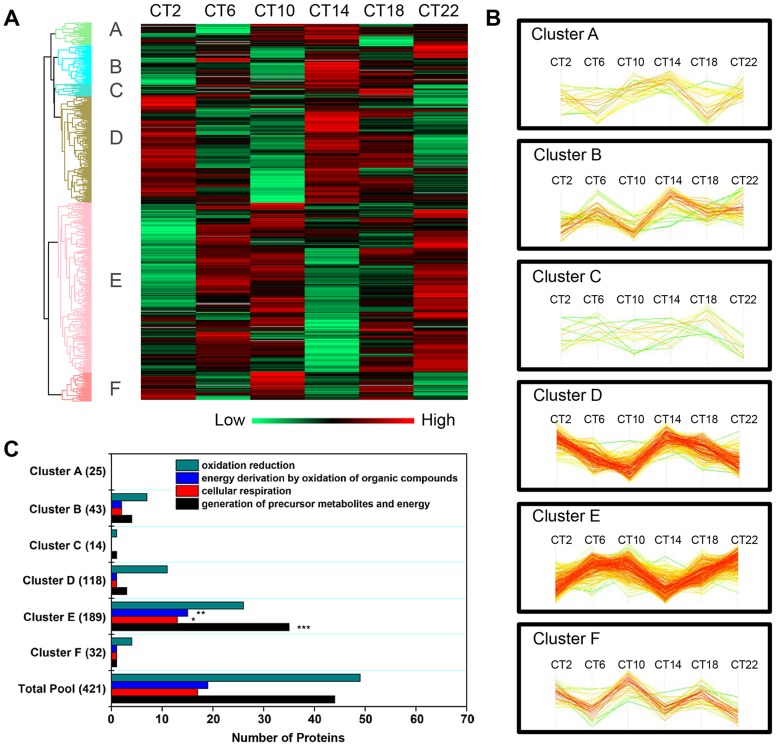
Cluster analysis of the time-of-day-dependent SCN proteome. (**A**) Hierarchical clustering of the 421 proteins that exhibited statistically significant, time-of-day-dependent expression in the SCN. After z-score normalization of the median value of logarithmized intensities (Log_2_) of each protein profile within Euclidean distances against those 421 time-of-day-dependent proteins, they were classified into six different expression clusters (denoted A through F). (**B**) Expression profile of the six hierarchical clusters, which were statistically different relative to one another. Two dominant clusters, B and E, were mirror images of one another. (**C**) Distribution of GO biological process terms in the six hierarchical clusters. Three GO biological processes were specifically enriched in cluster E relative to the time-of-day proteome. **p<0.01, ***p<0.001.

Hierarchical clustering of the time-of-day proteome revealed segregation of proteins into six different expression clusters (Figure 3A, Table S5). Two dominant clusters emerged from the analysis: cluster D (118 proteins) and cluster E (189 proteins). Closer examination of the protein levels within each cluster (Figure 3B) revealed that clusters D and E are mirror images of one another. A second interesting feature of clusters D and E is their bimodal expression profile. For instance, cluster E is characterized by an increase in expression from CT 2 to CT 10, a sharp decrease from CT 10 to CT 14 (the anticipated light-to-dark transition), a gradual increase through the night (CT 14 to CT 22), followed by an abrupt decrease from CT 22 to CT 2 (the anticipated dark-to-light transition). Interestingly, when compared to the time-of-day proteome, cluster E was selectively enriched for several GO biological processes such as generation of precursor metabolites and energy, cellular respiration, and energy derivation by oxidation of organic compounds (Figure 3C). Collectively, the data reveal that a substantial portion of the SCN proteome (20%) exhibits significant changes in abundance as a function of time-of-day.

### The Murine SCN Proteome: Existence of Ultradian Components and Relationship with the SCN Transcriptome

Next, we sought to identify proteins that exhibited a strictly circadian pattern of expression, as well as those that were ultradian. To this end, we employed the JTK_CYCLE algorithm [17], [18] to identify the subsets of proteins within the time-of-day proteome that oscillated with periods of 8, 12 and 24 h. JTK_CYCLE was recently developed to detect rhythmic components within large genomic datasets, and is superior to other similar algorithms in its sensitivity, specificity and efficiency [17]. Recent studies have also used JTK_CYCLE to analyze the circadian acetylome and the diurnal transcriptome of the murine liver and heart, respectively [19], [20]. Given that the observed free-running period of C57BL6 mice is ∼23.6 to 23.8 h, we used the nearest integer value (24) to approximate a circadian cycle using JTK_CYCLE. Based on a p-value cutoff of 0.05 [18], 48 proteins were deemed to be circadian, with phase of peak expression distributed across the entire 24 h cycle (Figure S2C, Table S6). Surprisingly, a relatively large proportion of the time-of-day proteome exhibited ultradian periods of 8 h (25 proteins) and 12 h (59 proteins) (p<0.05, JTK_CYCLE, Figures S2A and S2B, Table S7). Those 12 h rhythmic proteins tended to peak in expression at either the early day and early night, or late day and late night (Figure S2B), mirroring the profiles of clusters D and E (Figure 3B), respectively. Moreover, subjecting the larger dataset of the SCN proteome to JTK_CYCLE analysis resulted in the assignment of an additional 11, 41 and 43 proteins as rhythmic with periods of 8, 12 and 24 h, respectively. The fact that these proteins were identified as rhythmic by JTK_CYCLE but were not significantly altered based on ANOVA suggests that they might exhibit weak fluctuations that are mistaken as rhythmic. Thus, we focused subsequent downstream analyses on those circadian and ultradian proteins that were identified within the time-of-day proteome rather than the SCN proteome. Our results are somewhat reminiscent of the findings of Hughes et al. [18], which identified clusters of transcripts that cycled at the second and third harmonics of circadian rhythmicity in the murine liver; however, in that study the transcripts exhibiting these subharmonics accounted for only a small fraction of the entire rhythmic transcriptome.

**Figure 4:**
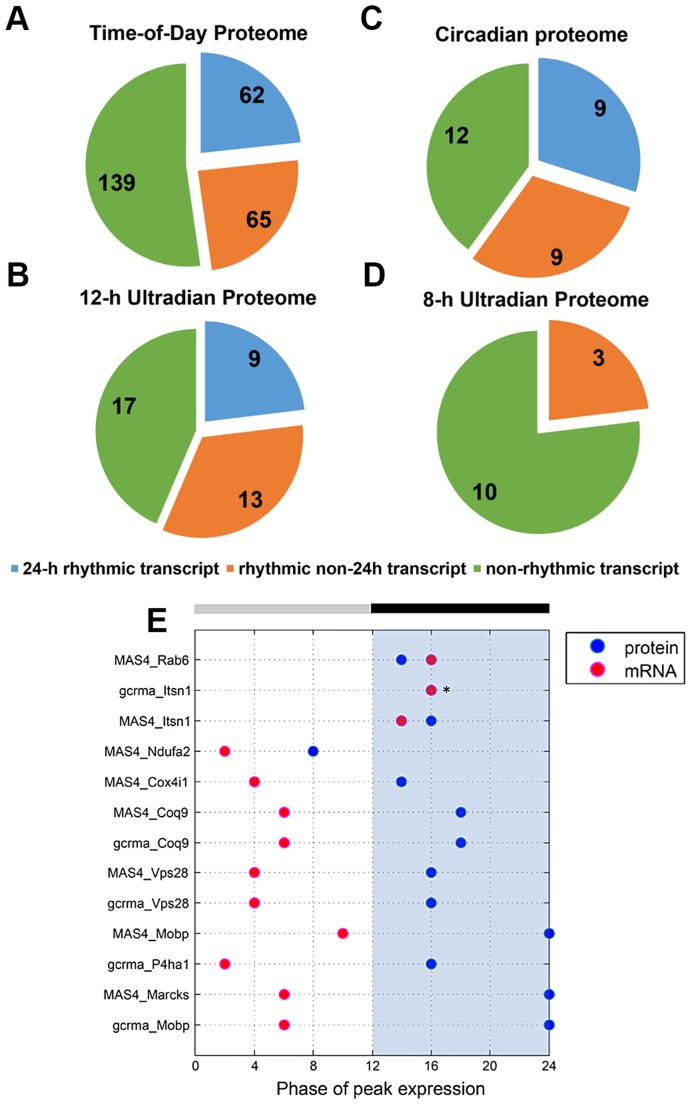
Comparative analysis of the murine SCN transcriptome and proteome. (**A–D**) Distribution of the (A) time-of-day proteome, (B) circadian proteome, (C) 12-h ultradian proteome, and (D) 8-h ultradian proteome according to profile of transcript expression. Transcript profile was classified as non-rhythmic (green), 24 h rhythmic (blue), or rhythmic non-24 h (orange). The rhythmic non-24 h transcripts oscillated at periods of either 16, 20, 28 or 32 h. Transcript data were acquired from two published microarray studies (MAS4 Panda et al and gcrma Panda et. al) of the mouse SCN transcriptome from the CIRCA database. Proteins without a corresponding transcript in CIRCA database are not represented in the pie charts. (**E**) Genes that are circadian at the transcript and protein level: a comparison of the phases of peak expression. Pink and blue dots represent the phase (CT) of peak expression at the mRNA and protein level, respectively. In general, expression of these 24 h rhythmic proteins lagged significantly behind expression of their corresponding transcripts. Asterisks (*) denote instances where the transcript and protein overlap in their phase of peak expression.

To investigate the relationship between transcript levels and protein abundance, we compared our time-of-day, circadian and ultradian (8 h and 12 h) proteomes with mRNA data extracted from two published microarray studies (MAS4 Panda et al. and gcrma Panda et al.) of the mouse SCN transcriptome from the CIRCA database (http://bioinf.itmat.upenn.edu/circa/) using identical JTK_CYCLE filtering criteria (p<0.05, 0 to 40 h period rhythmicity) (Figures 4A–D). Notably, >40% of each proteome was encoded by non-rhythmic transcripts. Circadian transcripts were found to encode a smaller subset of proteins within the time-of-day, circadian and 12-h ultradian proteomes. Each proteome also consisted of a subset of proteins that were encoded by rhythmic (non-24 h) transcripts cycling at intervals of 16, 20, 28 or 32 h. Notably, none of the 8 h and 12 h rhythmic proteins were encoded by transcripts that oscillated with the same period. Moreover, there were only 9 genes that exhibited circadian rhythms at both the transcript and protein level, and even amongst most of these there was a significant time lag (mean ∼8 h) between the peak in expression of the mRNA and the protein (Figure 4E).

Collectively, our data indicate that transcript levels are a generally poor predictor of protein abundance in the murine SCN. By extension, this suggests that post-transcriptional mechanisms play a dominant role in shaping the ultimate landscape of the SCN proteome. Another key observation from our study is that, for a substantial portion of the time-of-day proteome, the anticipated light-to-dark and dark-to-light transitions trigger robust changes in protein abundance that are similar in direction (either up- or down-regulated).

### The Potential Role of microRNAs in the Time-of-Day-Dependent SCN Proteome

**Figure 5:**
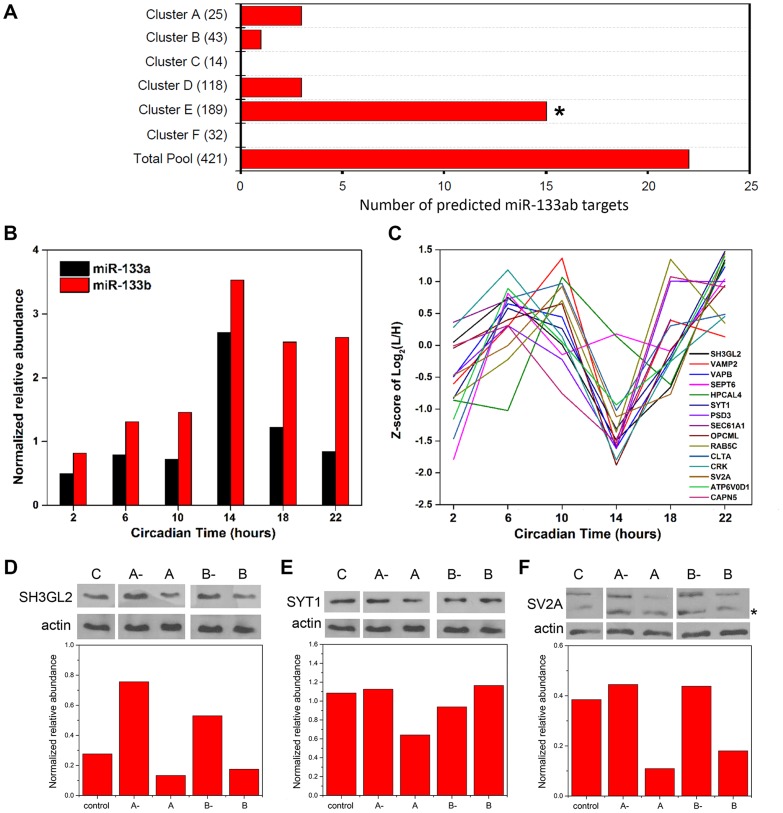
Functional implications for miRNA target enrichment in specific hierarchical clusters. (**A**) Distribution of the number of predicted miR-133ab murine target genes in the six hierarchical clusters. Compared to other hierarchical clusters, cluster E was significantly enriched for predicted murine targets of miR-133ab (Fisher's exact test, *p*<0.05). (**B**) Expression profiles of miR-133a and miR-133b, along with (**C**) those of their respective protein targets in cluster E. qRT-PCR was performed to detect and measure the relative abundance of miR-133a and miR-133b in the SCN. Information regarding predicted miRNA targets was extracted from the broadly conserved microRNA families from the TargetScanMouse database. (**D–F**) Western blot analysis of three predicted miR-133ab targets in cluster E, including (D) SH3GL2, (E) SYT1, and (F) SV2A, in Neuro2A cells transfected with either microRNA inhibitors against miR-133a (A-) or miR-133b (B-), microRNA mimics for miR-133a (A) or miR-133b (B), or microRNA inhibitor negative controls (C). Actin was used as the loading control in Western blot analysis. Values in each graph represent the median relative abundance of the protein examined normalized to actin expression (n = 3 per CT). The asterisk (*) denotes the presence of a faster-migrating, non-specific band.

microRNAs (miRNAs) are small (∼22–24 nt), noncoding RNAs that act as potent post-transcriptional modulators of gene expression. Various miRNAs have been implicated in the regulation of circadian rhythms in multiple model systems [21], [22]. To examine a possible involvement of miRNAs in shaping the SCN proteome, we first asked whether predicted targets of known miRNAs were enriched within particular hierarchical clusters of our time-of-day proteome. To this end, we compared the time-of-day proteome with the predicted targets of 86 broadly conserved miRNA families extracted from the TargetScanMouse version 6.2 database (http://www.targetscan.org/mmu_61/). Out of 86 broadly conserved miRNA families examined, only miR-133ab showed a significant enrichment of its respective targets in at least one hierarchical cluster. Figure 5A illustrates the number of predicted miR-133ab target genes within each hierarchical cluster based on the presence of at least one conserved site within the annotated 3' untranslated region (UTR) of the transcript. Compared to other hierarchical clusters, cluster E had the largest number of, and was statistically enriched for, predicted miR-133ab targets (Fisher's exact test, *p*<0.05; Figure 5A).

As miR-133ab levels have been reported to be low in the brain [23], we used an ultra-sensitive qRT-PCR approach to quantify levels of mature miR-133a and -133b in the murine SCN (Figure 5B). miR-133b abundance was elevated throughout the subjective night, whereas miR-133a levels peaked sharply at CT 14 (Figure 5B). Notably, the expression profile of miR-133ab showed an inverse trend when compared with the MS-quantified expression of their predicted target genes in cluster E from CT 10 to CT 22 (Figure 5C).

To provide functional evidence that these are authentic targets of miR-133ab, we selected three predicted targets within cluster E and examined their expression in Neuro2A cells in which levels of miR-133a or miR-133b have been enhanced using microRNA mimics, or suppressed by microRNA inhibitors. Either one or both mimics of miR-133a and miR-133b strongly suppressed the levels of SH3GL2, SYT1 and SV2A proteins in transfected Neuro2A cells compared with controls (Figures 5D–F). On the other hand, silencing of miR-133a or miR-133b robustly elevated the expression of SH3GL2 but not SYT1 or SV2A (Figures 5D–F). These data are consistent with SH3GL2, SYT1 and SV2A being authentic targets of miR-133ab.

Collectively, our results raise the possibility that miRNAs, such as miR-133ab, are orchestrating the temporal profiles of multiple target genes as suggested previously [24]. Given the fact that predicted miR-133ab targets are not solely restricted to a single hierarchical cluster with a common temporal expression pattern, other miRNAs are likely to work in concert with miR-133ab to fine-tune the temporal expression profile of its targets. More generally, our data suggest that miRNAs may be key post-transcriptional regulators of time-of-day-dependent protein expression within the SCN.

### Pathway Analysis of the SCN Proteome

**Figure 6:**
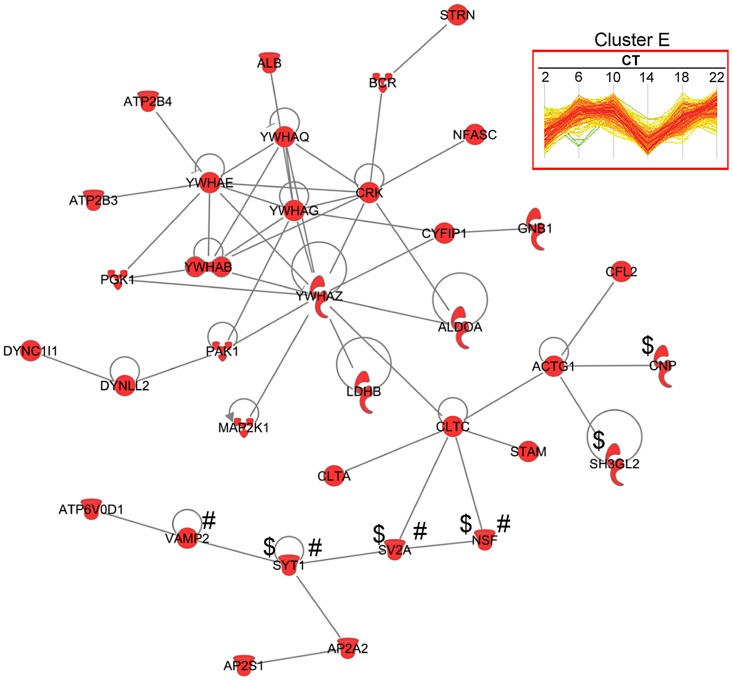
Protein interaction network of cluster E proteins. A total of 35 proteins from cluster E had direct protein-protein interactions based on IPA network analysis. Of note, proteins involved in neurotransmitter release (#) and synaptic transmission ($) were observed in this network.

The concerted expression of a large number of proteins within each hierarchical cluster suggests that many of these proteins might interact directly with one another to modulate a shared set of biological responses. To test this hypothesis, we used the IPA software to perform a very restrictive interaction analysis using only interactions from public repositories and limiting the network to direct interactions between proteins within a single cluster (i.e., no neighbors). Proteins in cluster E exhibited a high degree of connectivity (35 out of 189 proteins, or 18.5%) through direct protein-protein interactions (Figure 6). The top functions within this network were neurological disease and psychological disorders. Notably, this network included a relatively large number of proteins that are involved in neurotransmitter release (i.e., NSF, SV2A, SYT1 and VAMP2) and synaptic transmission (i.e., CNP, NSF, SV2A, SH3GL2 and SYT1) (Figure 6). A more comprehensive functional protein interaction network analysis of the entire time-of-day proteome (421 proteins) revealed that one of the largest networks is driven from the interactions among proteins involved in protein trafficking and carbohydrate metabolism (Figure S3). This network cluster included 9 proteins that oscillated with 12-h rhythms. The larger protein interaction network also included proteins involved in more general cellular functions, such as cellular assembly and organization, cellular function and maintenance, and cell morphology (Figure S3).

We further mapped the time-of-day proteome onto 192 known canonical pathways using IPA to identify pathways that might be significantly impacted (Figure S4). Three canonical pathways that had previously been implicated in the regulation of the SCN clock [25]–[27] were identified in our IPA analysis: Ca^2+^/cAMP response element binding protein (CREB) signaling in neurons (p = 0.0031); extracellular signal-regulated kinase (ERK)/mitogen- activated protein kinase (MAPK) signaling pathway (p = 0.0019); and synaptic long-term potentiation pathway (p = 0.0035).

**Figure 7:**
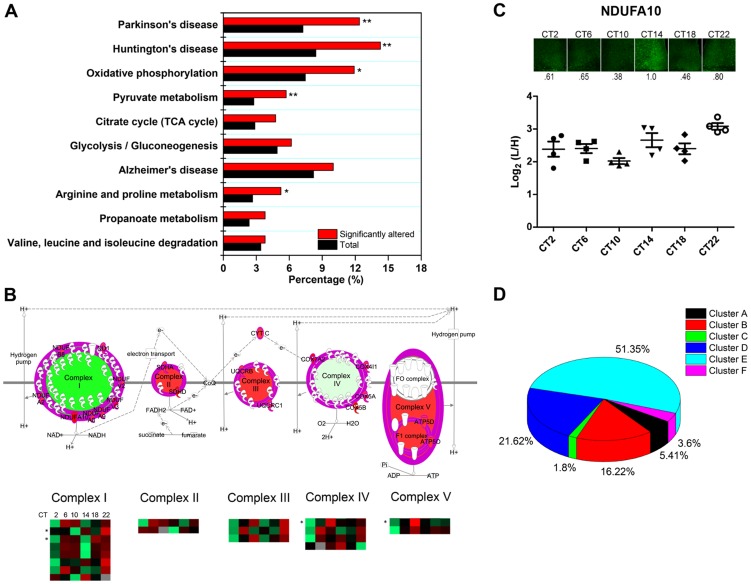
Mitochondrial oxidative phosphorylation represents a major axis of regulation within the SCN. (**A**) KEGG pathway enrichment analysis (by DAVID) of the time-of-day proteome. Pathways that are significantly enriched relative to the SCN proteome (2112 proteins) are denoted with an asterisk. *p<0.05, and **p<0.01 (Fisher's exact test). (**B**) Schematic representation of the 19 rhythmic proteins in our proteomic dataset that are involved in oxidative phosphorylation, based on IPA canonical pathway analysis. The asterisk (*) denotes four 24-h rhythmic proteins including NDUFA10, NDUFA2, COX4I1, and ATP5D. (**C**) Immunofluorescent (top) and MS (bottom) analysis of NDUFA10 expression in the SCN as a function of CT. IF images were acquired using a 20× objective. Values below each micrograph (top) represent the median relative abundance of NDUFA10 (n = 3 mice per CT). (**D**) Distribution of the 111 time-of-day-dependent mitochondrial proteins within the six hierarchical protein clusters.

In agreement with our previous GO analysis, the top-ranked canonical pathway was the mitochondrial dysfunction pathway, with 29 out of 421 proteins mapped. To delve further into the potential implications of an apparent temporal regulation of the mitochondria, we performed a KEGG pathway enrichment analysis by DAVID to compare our time-of-day proteome against all 2112 quantified proteins in the SCN proteome. Our data reveal that the KEGG pathways for Huntington's disease, Parkinson's disease, oxidative phosphorylation, pyruvate metabolism, and arginine and proline metabolism were significantly enriched with at least 8 proteins mapped in each pathway (Figure 7A). Indeed, many of the proteins within the Huntington's disease, Parkinson's disease, and oxidative phosphorylation (OxPhos) pathways overlapped and were mitochondrial in their localization. Particularly noteworthy was the OxPhos pathway, which accounted for 25 proteins within the time-of-day proteome. Of these 25 OxPhos-related proteins, 22 were present in cluster E and thus exhibited a similar trend in expression profile. Results from KEGG analysis mirrored those from canonical pathway analysis by IPA, which identified 19 OxPhos-related proteins (a subset of the 25), with 16 of these belonging in cluster E (Figure 7B). One of these OxPhos proteins, NDUFA10, a subunit of NADH∶ubiquinone oxidoreductase (complex I), was selected for validation by IF. NDUFA10 immunoreactivity within the SCN exhibited a pronounced increase at the CT10-CT14 transition, in keeping with the MS results at these two time points (Figure 7C).

The time-of-day proteome is significantly enriched for mitochondrial proteins (111/421, or 26.4%) relative to the SCN proteome (2112 proteins). Further analysis revealed that 51.4% of these mitochondrial proteins belonged within Cluster E, although this “cluster bias” did not reach statistical significance (Figure 7D). However, when we restricted our analysis to mitochondrial proteins that were involved in the OxPhos pathway, we found significant enrichment within cluster E. Our collective data point to a hitherto unappreciated temporal regulation of mitochondrial functions, particularly oxidative phosphorylation, within the central pacemaker.

## Discussion

Despite recent advances in quantitative proteomics and their application to the study of clock-controlled processes in the liver [14], [15], [19], the SCN proteome has been challenging to characterize in a comprehensive manner due to its inherently low sample availability. A previous attempt, using 2-dimensional difference gel electrophoresis (2D-DIGE) coupled with MS for protein identification, uncovered 115 proteins with time-of-day-dependent expression, of which 34 were circadian, out of 871 protein spots detected [7]. In our present study, we took advantage of the quantitative accuracy of SILAC, and combined it with the enhanced detection sensitivity that is achieved using the CPR, to provide a large-scale interrogation of the SCN proteome (Figure 1A). The outcome was the identification of 421 and 48 proteins whose expression profiles were time-of-day-dependent and circadian, respectively, from a stringently quantified dataset of 2112 proteins. In contrast with recently published liver circadian proteome studies [14], [15], we used 4 independent biological replicates to represent each CT across one 24 h cycle, rather than a single (pooled) sample for each time point across two cycles. Despite these procedural differences, the percentage of the detected proteome that exhibited circadian (24 h) rhythms was reasonably similar in all three studies (2.2%, our study; 6.0% Robles et al. [14]; 4.8% Mauvoisin et al. [15]). Furthermore, based on our proteomic analysis of the SCN, we were able to conclude that transcript expression is a relatively poor predictor of protein abundance, that ultradian rhythms in protein expression are prevalent in the SCN, and that the mitochondria, in particular oxidative phosphorylation, is a major target of temporal control in the central pacemaker. Additionally, our findings support the argument that post-transcriptional mechanisms, including miRNAs, may play a prominent role in shaping the ultimate landscape of the SCN proteome.

Comparisons with the Deery et al. report revealed that our SCN proteome (2112 proteins) included 30 of their 115 time-of-day-dependent proteins. Eleven out of these 30 were found within our time-of-day proteome and two belonged in our circadian proteome (p<0.05, JTK analysis). The higher frequency in sampling intervals in our study compared to theirs (4 h vs. 6 h) along with different statistical algorithms employed may partially account for the differences between the two datasets. Nevertheless, both studies identified vesicular trafficking and neurosecretory processes as novel points of temporal regulation. Interestingly, a number of proteins involved in neurotransmitter release (i.e., NSF, SV2A, SYT1 and VAMP2) and synaptic transmission (i.e., CNP, NSF, SV2A, SH3GL2 and SYT1) shared similar expression trends (cluster E), exemplifying how the SCN might coordinate the expression of proteins that act within the same biological pathway.

One unexpected observation was the sharp, and parallel, changes in protein expression at the anticipated transitions from light-to-dark and dark-to-light. This pattern was prevalent not only in the 12 h ultradian proteome (Figure S2B) but also in the time-of-day proteome (clusters D and E, Figure 3B). Hughes et al. [18] made similar observations in analyzing the 12 h cycling transcripts of the liver. They speculated that these dawn- and dusk-peaking transcripts took part in cellular mechanisms that anticipated the stress of daily transitions into light and darkness. Such an explanation might also be pertinent to our study. In particular, changes in levels of oxidative phosphorylation might constitute one such cellular response to light-dark/dark-light (or rest-wake/wake-rest) state transitions (Figure 7B). The mechanisms that drive these bimodal expression profiles within the SCN are unknown, but could conceivably involve interactions between the molecular clock and systemic cues. Alternatively, as shown by Westermark and colleagues [28], 12 h rhythms may arise theoretically through the actions of pairs of circadianly expressed transcription factors with defined phase relationships relative to each other.

Our study adds to the growing body of evidence that post-transcriptional regulation is a key feature of central and peripheral clocks. Less than 60% of the circadian and ultradian proteomes were encoded by transcripts that were rhythmic. In the case of the 8 h and 12 h proteomes, none of those rhythmic transcripts exhibited the same period as the encoded protein. Furthermore, for the 9 genes that were circadian at both the mRNA and protein level, there was a great disparity between the phase of peak expression of the transcript vs. that of the protein (mean time difference of ∼8 h). From these collective data, one can infer that post-transcriptional (and post-translational) regulation has a major influence on protein expression in the SCN. Specific post-transcriptional events, such as miRNA regulation, may intersect with circadian transcriptional rhythms (or even constitutive gene transcription) to establish the final time-course and profile of protein expression. Along these lines, we provide evidence that a microRNA, miR-133ab, has the ability to regulate the expression of various target proteins belonging to cluster E. Furthermore, we noted an inverse trend between miR-133ab levels and expression of their *in silico* targets in the SCN.

Another interesting finding is the prevalence of ultradian proteins in the SCN. Hughes et al. [18] also discovered circadian harmonics within his rhythmic transcriptome, but they occurred with a much lower frequency relative to the circadian transcripts. The mechanisms that are driving a larger subset of proteins towards ultradian expression are unclear, but could conceivably involve one or several post-transcriptional mechanisms.

Finally, our results strongly support the notion that mitochondrial energetics within the SCN is under strict temporal control (Figure 7A). While recent studies emphasize the circadian control of mitochondrial metabolism in the liver [19], [29], a key organ for energy storage and mobilization, almost none have directly studied mitochondrial function in the SCN. However, recent observations that the NAD^+^-dependent deacetylase SiRT1 positively modulates the expression of CLOCK and BMAL1 in the SCN [10], and that resveratrol activates SiRT1 through an increase in mitochondrial complex I-dependent NADH oxidation [30], raise the possibility that mitochondrial metabolism, in particular oxidative phosphorylation, may play a prominent role in maintaining robustness of the SCN clock. Interestingly, others have noted that hepatic NAD^+^ levels exhibit a bimodal rhythm and attributed this entirely to its biosynthesis by the enzyme nicotinamide phosphoribosyltransferase [31]. Our proteomics data, which highlight the bimodal expression of a large number of OxPhos-related proteins, provide an additional mechanism by which NAD^+^ levels may be shaped in the SCN.

In conclusion, our study provides a broader perspective on the temporal control of the SCN proteome. Our results underscore the significance of post-transcriptional regulation, the surprising prevalence of ultradian protein expression, and the functional implications on mitochondrial energy metabolism. Future investigations should help to clarify how each of these aspects contributes to the central pacemaker function of the SCN.

## Materials and Methods

### Ethics Statement

All animal handling and experimental procedures were conducted at the University of Toronto Mississauga animal facility, and were approved by the local animal care committee in compliance with institutional guidelines and the Canadian Council on Animal Care.

### Reagents

Ammonium bicarbonate (NH_4_HCO_3_), dithiothreitol (DTT), iodoacetamide (IAA), citric acid, and urea were obtained from Sigma-Aldrich (Saint Louis, MO). Acetonitrile, with 0.1% formic acid, and water, with 0.1% formic acid, were purchased from J.T. Baker (Phillipsburg, NJ). Trypsin was purchased from Promega (Madison, WI). Strong cation exchange (SCX) beads were obtained from Polymer Laboratories, Varian, Inc. (Palo Alto, CA). CHAPS (3-[(3-cholamidopropyl)dimethylammonio]-1- propanesulfonate, BP 571), ammonium hydroxide (NH_4_OH), and methanol were purchased from Fisher Scientific (Hampton, NH).

### Animals

Eight- to 12-week-old male C57BL6/J mice that were obtained from in-house breeding or purchase from The Jackson Laboratory (Bar Harbor, ME) were used for all experiments. Mice were group-housed in polycarbonate cages and given *ad libitum* access to rodent chow and water throughout the study.

### Tissue Harvest

Mice were stably entrained for a minimum of 2 weeks to a 12 h light∶12 h dark (LD) schedule (light intensity during the L phase was ∼200 lux) prior to transfer to complete darkness (DD) for 2 full cycles. Dark adaptation was achieved by placing cages into light-tight ventilated cabinets. On day 3 of DD, mice were killed at 4-h intervals at circadian times (CT) 2, 6, 10, 14, 18, and 22, where CT corresponds to the Zeitgeber time (ZT) of the previous LD cycle. Mice were killed by cervical dislocation and decapitated, and eyes were covered with black electrical tape under dim red light. Brains were dissected and cut into 800-µm thick coronal sections containing the SCN in cooled oxygenated media using an oscillating tissue slicer [24]. SCN was isolated from the tissue slice using a razor blade, and frozen immediately on dry ice. For IF, the entire coronal slice was fixed in 4% (w/v) paraformaldehyde in phosphate-buffered saline (PBS; 6 h, room temperature), cryoprotected in 30% (w/v) sucrose (overnight, 4°C), and cut into thin sections (30-µm) using a freezing microtome.

### Cell Culture

Neuro2A cells (American Type Culture Collection [ATCC], Manassas, VA) were grown in customized DMEM (AthenaES, Baltimore, MD, USA) and supplemented with [^13^C_6_,^15^N_4_]-L-Arginine (Arg-10), [^13^C_6_,^15^N_2_]-L-Lysine (Lys-8) at Arg 42 mg/L, Lys 146 mg/L, Met 30 mg/L and supplemented with 10% (v/v) dialyzed FBS (GIBCO-Invitrogen; Burlington, ON, Canada), 1 mM sodium pyruvate (Gibco-Invitrogen) and 28 µg/mL gentamicin (Gibco-Invitrogen). Labeled amino acids are purchased from Sigma-Aldrich (Oakville, ON, Canada). Cells were maintained in culture for at least 10 doubling times to allow for complete (>98%) incorporation of the isotope-labeled amino acids into the cells.

### SCN Proteomic Analysis by Centrifugal Proteomic Reactor

SCN tissues of individual mice were homogenized in 80 µL lysis buffer (8M urea, 4% CHAPS, 100 mM NH_4_HCO_3_ with fresh proteinase inhibitor mixture) in a 1.5 mL Pellet pestle, and sonicated 3 times for 10 s each with >30 s on ice between each pulse. Protein concentration was determined by the Bradford method. Proteins were processed in a centrifugal proteomic reactor device as previously described with some modifications [13]. Briefly, lysates from SCN tissues and heavy SILAC-labeled Neuro2A cells were mixed 1∶1 (30 µg protein each), and vortexed (1 min) vigorously in the presence of 30 µL of SCX slurry and 1.2 mL of 5% formic acid. Samples were centrifuged (16,100*×g*, 3 min), and SCX bead pellets were washed twice with 1.2 mL 0.1% formic acid. Proteins were reduced by incubating samples in the presence of 20 µL of 150 mM NH_4_HCO_3_, 20 mM DTT (1200 rpm, 56°C, 15 min), and were subsequently alkylated by the addition of 20 µL of 150 mM NH_4_HCO_3_, 100 mM IAA (room temperature, 15 min in darkness). The reaction was stopped by adding 1.2 mL of 0.1% formic acid supplemented with 6 µg trypsin. Following centrifugation, the SCX bead pellet was resuspended in 40 µL of 1 M NH_4_HCO_3_ and trypsin digested for 4 h (37°C, 1200 rpm). Finally, pH step elution of the peptides from the SCX beads was performed by adding: 1.2 ml of 0.1% formic acid pH 2.5, followed by additional nine pH fractions (pH 3.0, 3.5, 4.0, 4.5, 5.0, 5.5 6.0, 8.0, 12) by subsequent additions of 400 µL 10 mM citric acid/NH_4_OH pH buffers. The fractionated samples were desalted using in-house-made C_18_ desalting cartridges, and dessicated using a Speed-Vac prior to LC-MS measurement.

### LC-MS Measurement

All resulting peptide fractions were analyzed by HPLC-ESI-MS/MS, which consists of an automated Agilent 1100 micro-HPLC system (Agilent Technologies, Santa Clara, CA) coupled with an LTQ-Orbitrap mass spectrometer (ThermoFisher Scientific, San Jose, CA) equipped with a nano-electrospray interface operated in positive ion mode. Each peptide mixture was reconstituted in 20 µL of 0.5% (v/v) formic acid, and 10 µl was loaded on a 200 µm×50 mm fritted fused silica pre-column packed in-house with reverse phase Magic C18AQ resins (5 µm; 200-Å pore size; Dr. Maisch GmbH, Ammerbuch, Germany). The separation of peptides was performed on an analytical column (75 µm×10 cm) packed with reverse phase beads (3 µm; 120-Å pore size; Dr. Maisch GmbH, Ammerbuch, Germany). Gradient elution was performed over 75 min from 5–30% acetonitrile (v/v) containing 0.1% formic acid (v/v) at an eluent flow rate of 200 nL/min after in-line flow splitting. The spray voltage was set to +1.8 kV and the temperature of the heated capillary was 200°C. The instrument method consisted of one full MS scan from 400 to 2000 m/z followed by data-dependent MS/MS scan of the 10 most intense ions, a dynamic exclusion repeat count of 2, and a repeat exclusion duration of 90 s. The full mass was scanned in the Orbitrap analyzer with R = 60,000 (defined at *m/z* = 400), and the subsequent MS/MS analysis was performed in the LTQ analyzer. To improve the mass accuracy, all measurements in the Orbitrap mass analyzer were performed with on-the-fly internal recalibration (“Lock Mass”). The charge state rejection function was enabled, and single charge and unassigned charge ions were rejected. All data were recorded with the Xcalibur software (ThermoFisher Scientific, San Jose, CA).

### Database Search and Bioinformatic Analysis

Raw files were processed and analyzed by MaxQuant, Version 1.3.0.5 against the mouse International Protein Index protein sequence database (IPI Mouse, version 3.75), including commonly observed contaminants. The following parameters were used: cysteine carbamidomethylation was selected as a fixed modification; and the methionine oxidation and protein N-terminal acetylation were set variable modifications. Enzyme specificity was set to trypsin. Up to two missing cleavages of trypsin were allowed. SILAC double labeling (light: K0R0; heavy: K8R10) was set as the search parameter in order to assess the conversion efficiency. The precursor ion mass tolerances were 6 ppm, and fragment ion mass tolerance was 0.8 Da for MS/MS spectra. The false discovery rate (FDR) for peptide and protein was set at 1% and a minimum length of six amino acids was used for peptide identification.

The proteingroup file was imported into Perseus (version 1.3.0.4) for statistical analysis of the data. The raw dataset (3275 proteins) was filtered to include only proteins with a minimum peptide ratio count of 2 and with quantification values in a minimum of 12 of 24 MS measurements (or 24 independent SCN samples), resulting in a stringently quantified dataset of 2112 proteins. One-way ANOVA was used to analyze this stringent dataset for temporal regulation, with p-values<0.05 indicating statistical significance. For the hierarchical clustering analysis, median value of logarithmized values for the normalized L/H ratio of each protein profile was performed after z-score normalization of the data within Euclidean distances.

To identify the subset of 24-h rhythmic proteins, JTK_CYCLE algorithm [17] was used on the SCN proteomic (2112 proteins) or the time-of-day proteomic (421 proteins) dataset under R language. The JTK_CYCLE algorithm allows the user to input integer values when defining the (non-statistical) parameters of a search. The values for the ratio L/H normalized of each protein profile from 24 mice were used. Any missing values (i.e., not detected by MS) were replaced with zero prior to JTK_CYCLE analysis, as per expert recommendation (Dr. Michael Hughes, personal communication). Using a similar strategy as reported previously [32] to deal with missing values (ie., by replacing them with the minimum values observed for any given peptide) yielded the same results as replacing the missing values with zero, affirming the validity of our approach. p-values (ADJ.P) less than 0.05 were considered significant, and the corresponding proteins were classified as displaying a circadian rhythm [14]. To find the 8-h or 12-h rhythmic proteins within the 421 protein dataset, another JTK_CYCLE analysis was separately performed with period lengths set at 8 and 12-h, respectively.

Canonical pathways analyses and protein network of the time-of-day proteomic (421 proteins) dataset were mapped and summarized by Ingenuity Pathways Analysis (IPA), version 8.5 (Ingenuity Systems, Redwood City, CA). Canonical pathways analyses were performed with p value of 0.05 and networks were displayed with minimum significant score of 16. Kyoto Encyclopedia of Genes and Genomes (KEGG) pathway analysis was achieved using the DAVID Bioinformatics Resources (http://david.abcc.ncifcrf.gov). DAVID statistical analyses are performed against the whole genome [12]. Proteomics has a tendency to oversample proteins from the cytosol while undersampling nuclear and membrane-associated proteins. To calculate exactly significant enrichment of time-of-day proteome in each GO term, we first calculated the amounts of matched proteins enriched either in the time-of-day proteome (421 proteins) or the total SCN proteome (2112 proteins). Fisher's exact test was used to check that the GO results were significantly enriched in the time-of-day proteome relative to the total SCN proteome. This was done to avoid any pathway/GO enrichment biases that would result from comparing our time-of-day proteome against the whole mouse database.

The mass spectrometry proteomics data have been deposited to the ProteomeXchange Consortium (http://www.proteomexchange.org) via the PRIDE partner repository [33] with the dataset identifier PXD000778.

### Real-Time PCR

Total RNA was extracted from individual SCN tissues using the Trizol Reagent according to manufacturer's instructions. RNA concentration and purity were determined using the NanoPhotometer P-Class (Implen GmbH, Germany), and RNA integrity was confirmed by agarose gel electrophoresis. cDNA synthesis was performed using the Universal cDNA Synthesis Kit II (Exiqon) and 20 ng of total RNA (for miR-133b) or 100 ng of total RNA (for miR-133a). cDNA was diluted 1∶40 and real-time PCR was performed using the ExiLENT SYBR Green Master Mix (Exiqon) and hsa-miR-133a and hsa-miR-133b LNA™ PCR primer sets (Exiqon), on the Stratagene Mx3000P qPCR System. Values were normalized to 18S ribosomal RNA abundance.

### *In Vitro* miRNA Mimic and Inhibitor Experiments

Neuro2A cells were grown on 6-well plates in DMEM containing 5% FBS and 1% penicillin-streptomycin at 37°C and 5% CO_2_ until they reached 75–80% confluence. Cells were then transfected in duplicate using Lipofectamine 2000 (Invitrogen) according to manufacturer's instructions. Cells were transfected with one of the following: miRCURY LNA Power Inhibitor (Exiqon) targeted towards either miR-133a or miR-133b; miRCURY LNA microRNA Mimic for either miR-133a or miR-133b; or miRCURY LNA microRNA inhibitor negative control. Protein lysates were harvested 24 h post-transfection for Western blot analysis.

### Western Blot Analysis

SCN containing tissues were homogenized on ice in RIPA buffer containing protease inhibitors. Homogenized tissues were incubated on ice for 20 min and centrifuged at 4°C at 17,000*× g* for 20 min. The supernatant was collected and stored at −80°C for downstream analysis. Protein concentration was measured using the Bradford assay. Protein lysates were mixed with SDS loading buffer to 1× concentration, heated at 95°C for 5 min, and centrifuged for 1 min at 17,000× g. Lysates (20 µg/well) were electrophoresed in a SDS polyacrylamide gel for approximately 2 h at 100 V at room temperature (RT) and electroblotted onto polyvinylidene fluoride (Immobilon P; Millipore, Bedford, MA) membrane for either 1 h at RT at 85 V, or overnight (O/N) at 4°C at 30 V. Protein transfer was confirmed using Ponceau S, followed by 3 washes for 5 min each in Tris Buffered Saline with 0.1% Triton X-100 (TBS-T). Membranes were blocked in 5% skim milk in TBS-T for 1 h at RT, followed by O/N incubation at 4°C with one of the following primary antibodies in blocking solution: rabbit anti-SYT1 (1∶500; cat #3347; Cell Signaling Technologies); rabbit anti-SV2A (1∶500; cat #ab32942; Abcam); rabbit anti-VAMP2 (1∶2000; cat#13508; Cell Signaling Technologies); rabbit anti-SH3GL2 (1∶2000; cat#ab169762; Abcam); rabbit anti-PAK1 (1∶500; cat#40852; Abcam); and rabbit anti-actin (1∶10,000; Sigma-Aldrich). The next day, membranes were washed in TBS-T and incubated for 2 h at RT with goat anti-rabbit horseradish peroxidase (HRP) conjugated secondary antibody (1∶250,000; ThermoFisher Scientific) in blocking solution. Chemiluminescent signal was developed using the SuperSignal West Femto Maximum Sensitivity Substrate reagent (ThermoFisher Scientific). Quantitation of western blots performed using the “measure” function in ImageJ (http://rsbweb.nih.gov/ij/) yielded a “mean gray” value for each protein band, which were normalized to background “mean gray” values. Values are presented as median relative abundance of the protein examined normalized to relative abundance of actin from 3 mice per time point.

### Immunofluorescence

Tissue sections were washed 5 times for 5 min each in Phosphate Buffered Saline with 0.1% Triton X-100 (PBS-T). Sections were blocked in 10% horse serum in PBS-T for 1 h at RT and incubated O/N with one of the following primary antibodies in blocking solution: rabbit anti-NDUFA10 (1∶1000; cat #ab103026; Abcam). The next day tissues were washed 5×5 min in PBS-T, and incubated for 2 h at RT in the dark with Alexa Fluor 488 donkey anti-rabbit secondary antibodies (1∶1000; Invitrogen) in blocking solution. Sections were washed 5×5 min in PBS-T, incubated with DAPI for 5 minutes, and washed with PBS. Sections were mounted on microscope slides, cover-slipped with fluorescence DAKO mounting medium and sealed with nail-polish. Slides were stored at 4°C.

### Imaging

IF images were captured using a Zeiss Axio Observer Z1 inverted microscope equipped with a Laser Scanning Microscope (LSM) 700 module along with the ZEN 2010 software (Zeiss, Oberkochen, Germany). Individual fluorochrome signals were collected sequentially using the multitrack setting along with appropriate barrier filters using the “smart set-up” option. IF images were acquired from a central focal plane of 2.3 µm optical thickness using the 10×, 20× or 40× objectives, or 1.0 um optical thickness using the 63× objective. Identical settings for gain, pinhole size, and brightness were used to acquire all images of the same magnification within each experiment. Adjustments to brightness and contrast were applied equally to all images within an experiment using Adobe Photoshop CS.

For quantitative analysis, the bilateral SCN from the micrographs were outlined using the polygon tool in ImageJ. The “measure” function yielded a “mean gray” value for each of the two bilateral SCN, which were normalized to background “mean gray” values obtained from surrounding non-immunoreactive hypothalamalic regions. Values are presented as median relative abundance of the protein examined from 3 mice per time point.

## Supporting Information

Figure S1 FAT Gene Ontology enrichment analysis of the time-of-day proteome. (A–C) GO FAT analysis of 421 time-of-day-dependent proteins (significantly altered: blue bars) and 48 circadian proteins (red bars) for (A) cellular component, (B) biological process, and (C) molecular function by DAVID. Black bars represent the total SCN proteome of 2112 proteins. GO FAT analysis confirmed that a significant proportion of the time-of-day-dependent proteins were associated with the mitochondrion, energy generation and consumption, and hydrogen ion transmembrane transporter activity. *p<0.05, **p<0.01, and ***p<0.001.
(TIF)

Figure S2 Heat maps of ultradian and circadian proteins within the time-of-day proteome. (A–C) Hierarchical clustering of (A) 8-h rhythmic, (B) 12-h rhythmic and (C) 24-h rhythmic proteins (p<0.05, JTK_cycle analysis) within the time-of-day proteome. The color of spots corresponds to the value for each protein (in rows) for each of 24 SCN samples (in columns) based on the logarithmized values of L/H normalized ratios after z-score normalization. (Larger than zero: red, green: smaller than zero, grey: NaN, not detected).
(TIF)

Figure S3 Protein interaction networks of the time-of-day proteome. A comprehensive functional protein interaction network analysis of the time-of-day proteome revealed that several diseases and functions were connected and enriched in the SCN proteome by using IPA software. Proteins labeled in green, orange, or pink indicate those which belong to the 8 h, 12 h or 24 h proteome group, respectively.
(TIF)

Figure S4 Canonical pathway analysis by IPA of the time-of-day proteome. Enriched canonical pathways observed in the time-of-day proteome (*p*<0.05). and three canonical pathways previously implicated in the regulation of the SCN clock were identified. In addition, the top-second ranked canonical pathway was the oxidative phosphorylation pathway, with 19 out of 421 proteins mapped, in agreement with our previous KEGG analysis by DAVID. Interestingly, the “breast regulation by stathmin pathway” was also identified, due to the presence of 15 proteins (CAMK1, GNAQ, TUBA4A, HRAS, KRAS, CDK1, GNB1, STMN1, PAK1, CAMK2A, RRAS2, TUBA1A, RHOA, TUBB4A, and MAP2K1) that mapped to this pathway but that are also involved in more general functions of the cell.
(TIF)

Table S1 Total identified protein dataset in the murine SCN. The list of 3275 identified proteins resulting from the proteinGroups of MaxQuant analysis of mass spectrometry RAW files. Following analysis using the Perseus software, values for the ratio L/H normalized (where L and H represent the normalized intensities of SCN and N2A lysates for each identified protein) for each biological sample (24 total, n = 4 per CT, 6 different CT) are presented in columns A to X. Column Y shows the order of proteins which exhibit statistically significant (1–421), or non-significant (422–3275) expression within a 24-h cycle. A total of 7 proteins (3269–3275) lacked a corresponding SILAC-labeled peak, indicating that these proteins are expressed in the SCN but not in Neuro2A cells.
(XLSX)

Table S2 The SCN proteome. The list of 2112 accurately quantified proteins representing the SCN proteome. This list was generated by filtering the raw protein dataset (3275 identified proteins) for only those proteins which were identified by a minimum of two peptide ratio counts and for which accurate quantification values were obtained in a minimum of 12 out of 24 independent samples.
(XLSX)

Table S3 Pearson r correlation values of the 2112 proteins in the SCN proteome. Pairwise Pearson's correlation analysis of 24 measurements using the SCN proteome dataset (2112 proteins).
(XLSX)

Table S4 Pearson r correlation values of the 421 proteins in the time-of-day proteome. Pairwise Pearson's correlation analysis of 24 measurements using the time-of-day proteome dataset (421 proteins) revealed that the r values of biological replicates at a specific CT were higher than between samples of different CTs.
(XLSX)

Table S5 The time-of-day proteome. The time-of-day proteome is comprised of 421 significantly altered proteins, segregated into six different expression clusters. Columns AK to AP indicate the median value of logarithmized values for the normalized L/H ratio of each protein profile in six CTs after z-score normalization of the data within Euclidean distances. Column AQ indicates the results of hierarchical clustering.
(XLSX)

Table S6 The circadian proteome of the murine SCN. Within the time-of-day proteome, a total of 48 proteins were considered to be circadian (24-h rhythmic) based on a JTK p-value cutoff of 0.05. Column AL shows the p-value (ADJ.P). Columns AM, AN and AO indicate the period (PER), optimal phase (LAG), and amplitude (AMP) estimates, respectively, for each protein according to JTK_CYCLE algorithm analysis.
(XLSX)

Table S7 List of 8 h and 12 h rhythmic proteins in the murine SCN. Within the time-of-day proteome, 25 and 59 proteins were characterized as either 8-h or 12-h rhythmic, respectively, based on a JTK p-value cutoff of 0.05.
(XLSX)
