# Supplementary figures and images for: The Proteomic Landscape of the Suprachiasmatic Nucleus Clock Reveals Large-Scale Coordination of Key Biological Processes
Source: PLoS Genet. 2014 Oct 16;10(10):e1004695. doi: 10.1371/journal.pgen.1004695 (PMC4199512; doi:10.1371/journal.pgen.1004695)

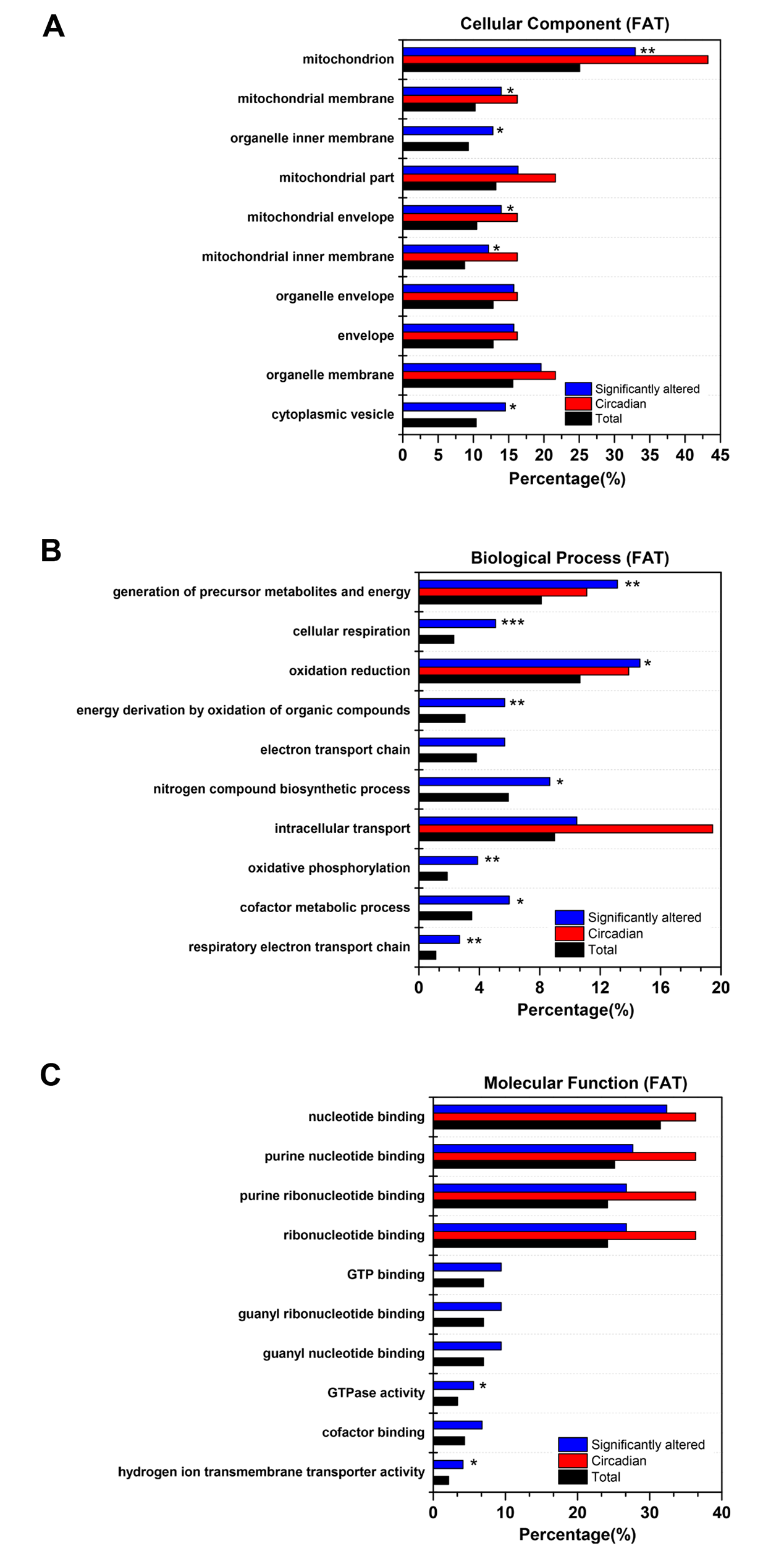

Supplement: Figure S1 — FAT Gene Ontology enrichment analysis of the time-of-day proteome. (A–C) GO FAT analysis of 421 time-of-day-dependent proteins (significantly altered: blue bars) and 48 circadian proteins (red bars) for (A) cellular component, (B) biological process, and (C) molecular function by DAVID. Black bars represent the total SCN proteome of 2112 proteins. GO FAT analysis confirmed that a significant proportion of the time-of-day-dependent proteins were associated with the mitochondrion, energy generation and consumption, and hydrogen ion transmembrane transporter activity. *p<0.05, **p<0.01, and ***p<0.001. (TIF) [file pgen.1004695.s001.tif]

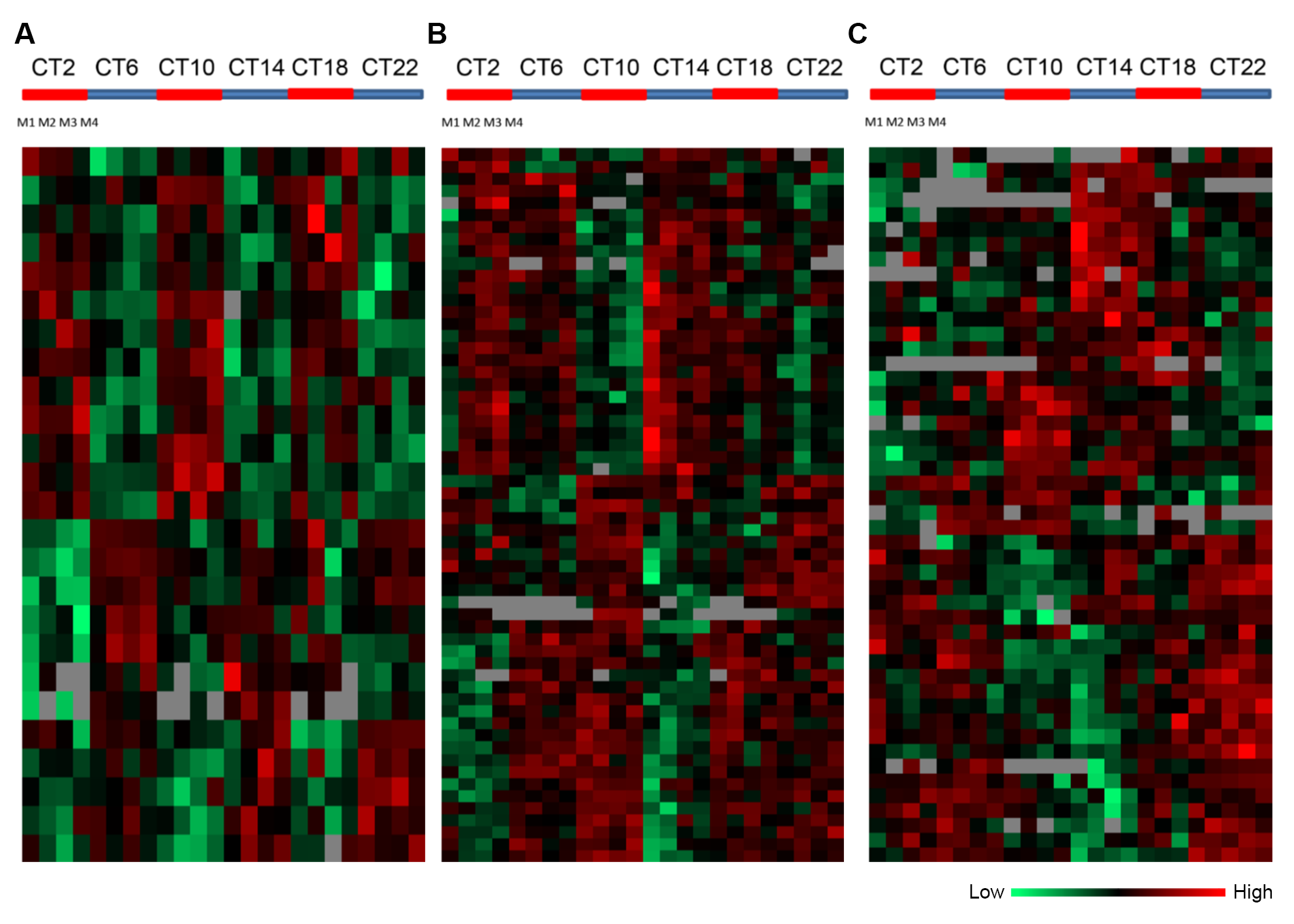

Supplement: Figure S2 — Heat maps of ultradian and circadian proteins within the time-of-day proteome. (A–C) Hierarchical clustering of (A) 8-h rhythmic, (B) 12-h rhythmic and (C) 24-h rhythmic proteins (p<0.05, JTK_cycle analysis) within the time-of-day proteome. The color of spots corresponds to the value for each protein (in rows) for each of 24 SCN samples (in columns) based on the logarithmized values of L/H normalized ratios after z-score normalization. (Larger than zero: red, green: smaller than zero, grey: NaN, not detected). (TIF) [file pgen.1004695.s002.tif]

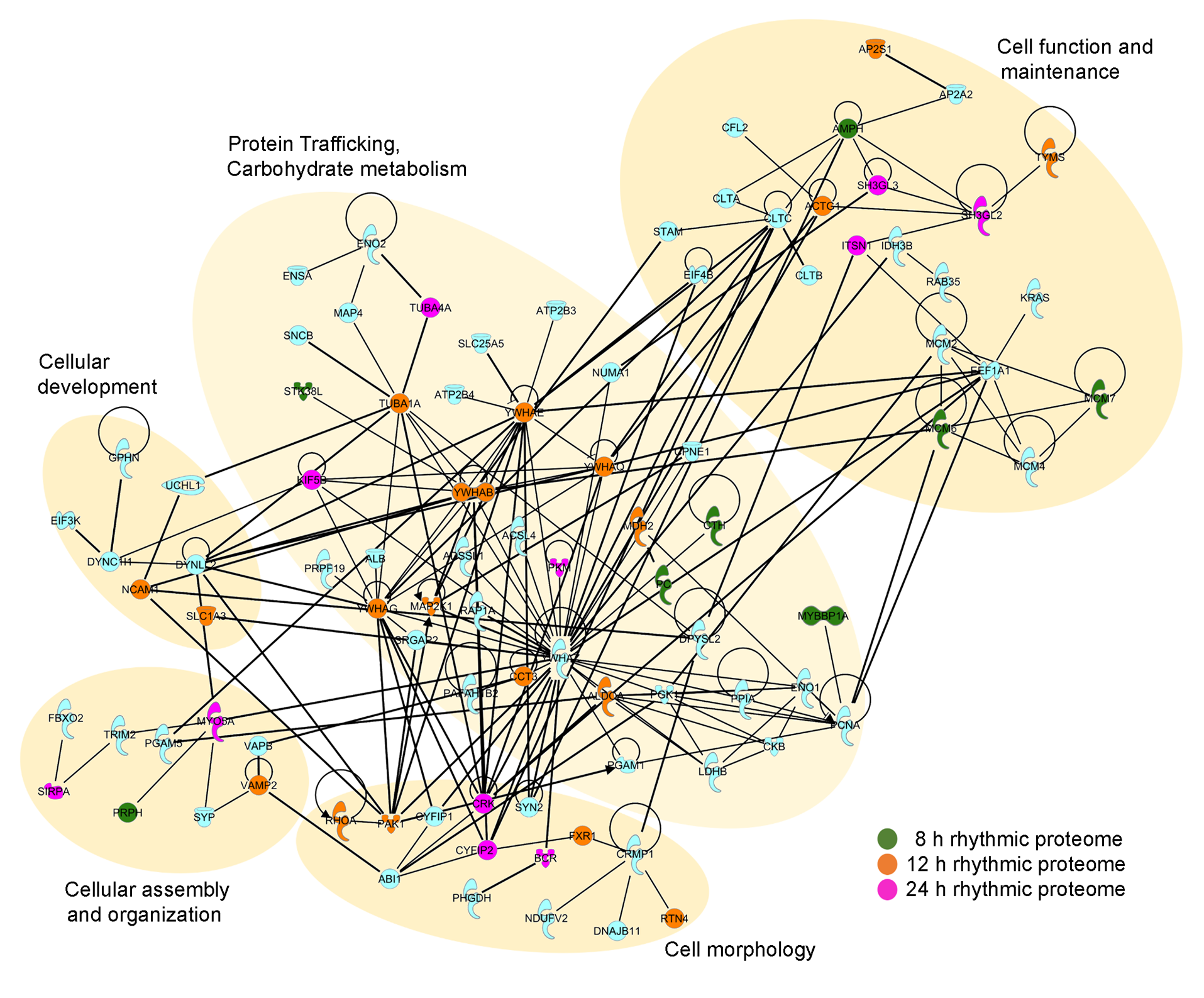

Supplement: Figure S3 — Protein interaction networks of the time-of-day proteome. A comprehensive functional protein interaction network analysis of the time-of-day proteome revealed that several diseases and functions were connected and enriched in the SCN proteome by using IPA software. Proteins labeled in green, orange, or pink indicate those which belong to the 8 h, 12 h or 24 h proteome group, respectively. (TIF) [file pgen.1004695.s003.tif]

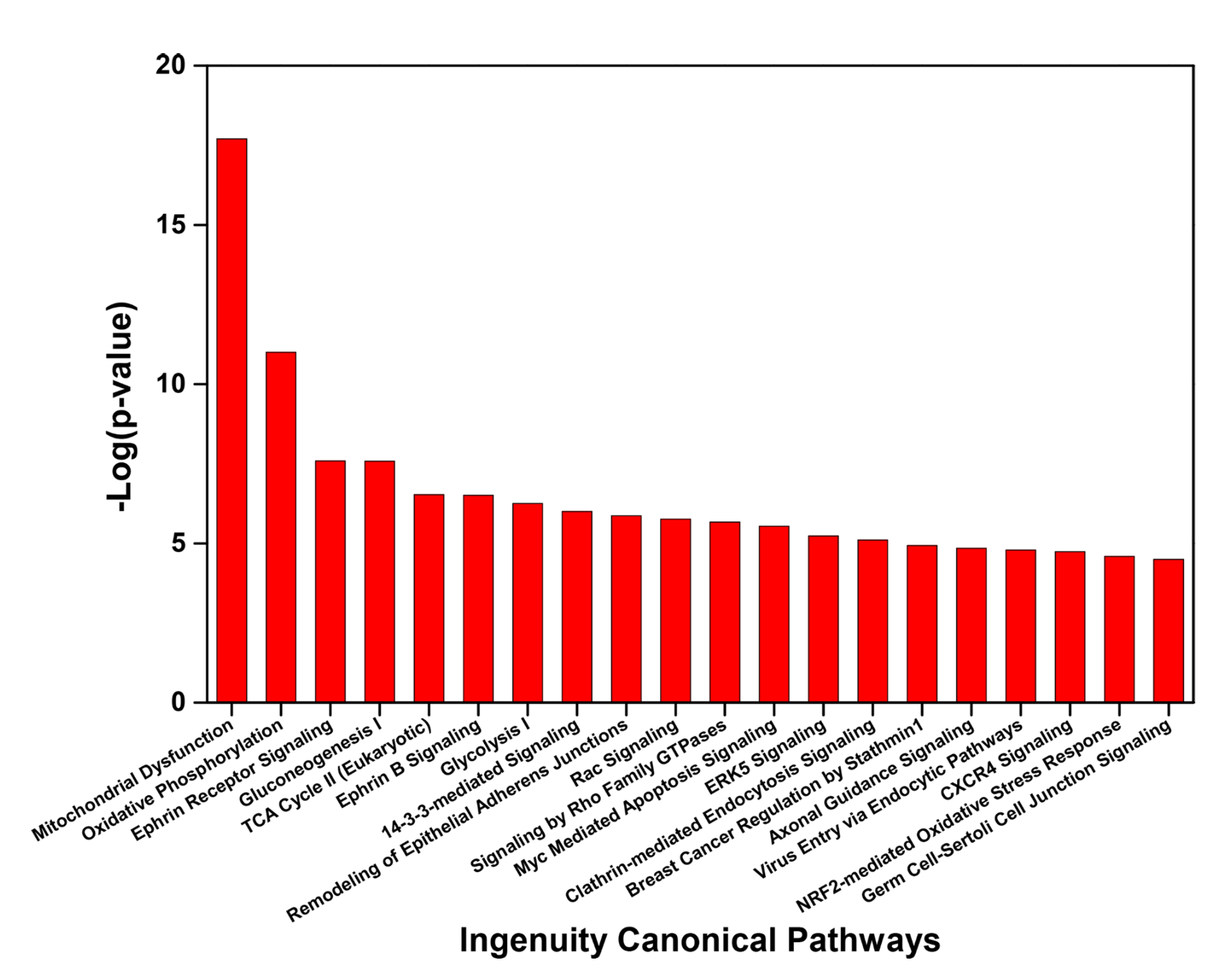

Supplement: Figure S4 — Canonical pathway analysis by IPA of the time-of-day proteome. Enriched canonical pathways observed in the time-of-day proteome (p<0.05). and three canonical pathways previously implicated in the regulation of the SCN clock were identified. In addition, the top-second ranked canonical pathway was the oxidative phosphorylation pathway, with 19 out of 421 proteins mapped, in agreement with our previous KEGG analysis by DAVID. Interestingly, the “breast regulation by stathmin pathway” was also identified, due to the presence of 15 proteins (CAMK1, GNAQ, TUBA4A, HRAS, KRAS, CDK1, GNB1, STMN1, PAK1, CAMK2A, RRAS2, TUBA1A, RHOA, TUBB4A, and MAP2K1) that mapped to this pathway but that are also involved in more general functions of the cell. (TIF) [file pgen.1004695.s004.tif]
